# Polyenes in Medium Chain Length Polyhydroxyalkanoate (mcl-PHA) Biopolymer Microspheres with Reduced Toxicity and Improved Therapeutic Effect against *Candida* Infection in Zebrafish Model

**DOI:** 10.3390/pharmaceutics14040696

**Published:** 2022-03-24

**Authors:** Aleksandar Pavic, Zoran Stojanovic, Marina Pekmezovic, Đorđe Veljović, Kevin O’Connor, Ivana Malagurski, Jasmina Nikodinovic-Runic

**Affiliations:** 1 Institute of Molecular Genetics and Genetic Engineering, University of Belgrade, 11042 Belgrade, Serbia; sasapavic@imgge.bg.ac.rs (A.P.); ivana.malagurski@gmail.com (I.M.); 2 Institute of Technical Sciences of the Serbian Academy of Sciences and Arts, 11000 Belgrade, Serbia; zoran.stojanovic@itn.sanu.ac.rs; 3 Junior Research Group Adaptive Pathogenicity Strategies, Leibniz Institute for Natural Product Research and Infection Biology, Hans Knöll Institute, 07745 Jena, Germany; marina.pekmezovic@hki-jena.de; 4 Faculty of Technology and Metallurgy, University of Belgrade, 11000 Belgrade, Serbia; djveljovic@tmf.bg.ac.rs; 5 BiOrbic Bioeconomy SFI Research Centre, Belfield, D04 V1W Dublin, Ireland; kevin.oconnor@ucd.ie; 6 School of Biomolecular and Biomedical Sciences, University College Dublin, Belfield, D04 V1W Dublin, Ireland

**Keywords:** polyene, polyhydroxyalkanoate, PHA, formulation, microsphere, *Candida albicans*, zebrafish

## Abstract

Immobilizing antifungal polyenes such as nystatin (Nys) and amphotericin B (AmB) into biodegradable formulations is advantageous compared to free drug administration providing sustained release, reduced dosing due to localized targeting and overall reduced systemic drug toxicity. In this study, we encapsulated Nys and AmB in medium chain length polyhydroxyalkanoates (mcl-PHA) microspheres (7–8 µm in diameter). The obtained formulations have been validated for antifungal activity in vitro against a panel of pathogenic fungi including species of *Candida*, *Aspergillus*, *Microsporum* and *Trichophyton* genera and toxicity and efficacy in vivo using the zebrafish model of disseminated candidiasis. While free polyenes, especially AmB, were highly toxic to zebrafish embryos at the effective (MIC) doses, after their loading into mcl-PHA microspheres, inner organ toxicity and teratogenicity associated with both drugs were not observed, even at 100 × MIC doses. The obtained mcl-PHA/polyene formulations have successfully eradicated *C. albicans* infection and showed an improved therapeutic profile in zebrafish by enhancing infected embryos survival. This approach is contributing to the antifungal arsenal as polyenes, although the first broad-spectrum antifungals on the market are still the gold standard for treatment of fungal infections.

## 1. Introduction

Disseminated candidiasis is the fourth leading infection in hospitalized patients in the United States and Europe, surpassing many bacterial pathogens, with the mortality rates remaining disturbingly high at 40% [1,2]. Fungal infections are commonly treated with five main classes of antimicrobial agents: (i) azoles, (ii) echinocandins, (iii) polyenes, (iv) allylamines and (v) pyrimidine analogs [3,4]. However, besides problems related to the fungal resistance, there are some limitations in their clinical application: (i) issues with drug safety profiles, (ii) pharmacokinetic properties, (iii) side effects, (iv) limited activity spectrum, (v) small number of available targets and (vi) no new antifungal drugs have been introduced in the market since 2006 when the European Medicines Agency (EMA) and the Food and Drug Administration (FDA) approved anidulafungin [3,5].

Polyenes represent the oldest family of antifungal drugs, available on the market for more than 60 years [6] and they are a class of bioactive antifungal molecules still being isolated and characterized from novel bacterial species [7]. Despite its toxic nature, amphotericin B (AmB) is considered as the “gold standard” in the treatment of many systemic fungal infections, mostly due to broad spectrum of activity and low resistance rate [6,8]. Nystatin (Nys) is one of the most commonly used topical antifungal drugs for treatment of oral or vaginal candidiasis. Some studies have reported that Nys prophylaxis prevented invasive candidiasis in preterm infants [9,10]. However, the dose-related toxicity of free drugs, especially AmB, limits their parenteral application and clinical success in treating fungal infections. Therefore, novel formulations of both polyene drugs are needed in order to minimize their toxicity, reduce the costs and improve the efficiency.

Polyhydroxyalkanoates (PHAs) are a family of biopolymers synthesized by bacteria as carbon and energy storage materials. They vary in their physical characteristics based on the monomer composition with short chain length (scl) PHAs being hard brittle polymers while medium chain length (mcl) PHAs are more elastomeric soft polymers [11]. PHAs are extensively studied for biomedical applications due to their desirable properties such as biocompatibility, biodegradability, mechanical strength and their capacity to be functionalized. The wide range of biomedical applications includes drug delivery systems [12,13,14,15], implants and tissue engineering scaffolds [16,17]. Given that pronounced toxicity of polyenes limits systemic use of these generally effective antifungal drugs and considering the general lack of new antifungal agents, the objective of this study was to produce new biopolymer-based formulations of antifungal polyenes for controlled delivery and improved safety in potential therapeutic applications. To achieve this, standardly used polyenes (Nys and AmB) were immobilized into biocompatible and biodegradable matrix (mcl-PHA). The obtained formulations were assessed in terms of immobilized drug efficacy and toxicity using the zebrafish model for treatment of candidiasis.

## 2. Materials and Methods

### 2.1. Mcl-PHA/Polyene Microsphere Synthesis

Mcl-PHA, in the form of a copolymer polyhydroxyoctanoate-co-decanoate (P3(HO-co-DO) with equimolar amount of hydroxyoctanoate and hydroxydecanoate monomers, was produced and characterized as in our previous study [18]. Mcl-PHA microspheres were prepared using solvent evaporation technique from double emulsions as reported before [19], with some modifications. Solution A, 0.5 wt % PVA 88000 MW (Acros Organics, 88%) was prepared and aged for 24 h at 25 °C. Solution B was prepared by dissolving 200 mg of mcl-PHA in chloroform (5 mL), followed by filtration and mixing with N-Methyl-2-pyrrolidone (NMP) (8 mL) on a magnetic stirrer and in an ultrasonic bath. Solution B was added drop wise into a beaker with 50 mL of solution A while stirring at 1400 rpm for 2 min. Freshly prepared emulsion was added to a larger beaker with 200 mL of gently stirred solution A at 30 °C and continued to stir for 5 h. Obtained colloidal solution was left to age in the refrigerator at 4 °C for 24 h. The supernatant was decanted and obtained concentrated cold dispersion was dried for 2 h under the pressure of 15 mbar to remove residual chloroform. Dried dispersion was centrifuged at 2000 rpm at 1 °C for 30 min and washed with distillated water to remove PVA and NMP residues. This was repeated 4 times. Separated mcl-PHA microspheres precipitate was then mixed with 0.5 mL 10 wt % water solution of L–mannitol and frozen at −10 °C for 24 h. Frozen suspension was dried to obtain a powder. To produce polyene-loaded microspheres, a similar procedure was applied using solution B with polyenes (Acros Organics, Geel, Belgium). Appropriate amounts of either Nys or AmB were dissolved in NMP before addition to PHA/chloroform solution to obtain microspheres with 10 and 20 wt% polyene content.

### 2.2. Characterization of Microspheres

Morphology of the samples was observed by a TESCAN MIRA 3 XMU field emission scanning electron microscope (FE-SEM), operated at 20 keV. Prior to analysis, freshly prepared microspheres were washed, re-dispersed in water–ethanol (50:50) at 0 °C, added drop-wise onto medium permeable filter paper, freeze dried and coated with gold.

To investigate in vitro polyene release from the samples, microspheres (50 mg) with 20% (*w*/*w*) polyene loading were incubated in glass bottles (100 mL volume) with 50 mL of 10 mM phosphate buffer (pH 7) in a shaking water bath (120 rpm) at 37 °C. At specific time intervals (0, 1, 2, 6, 12, 24, 30, 48, 72 and 96 h) 5 mL samples were taken and replaced with 5 mL of fresh release medium. Withdrawn samples were filtered through the Whatman filter paper and assayed spectrophotometrically for Nys and AmB content in triplicate by an UV/VIS spectrophotometer (Shimadzu 1800) at the maximum absorption wavelength, λmax, 305 and 416 nm, respectively [20,21], using a standard calibration curve.

Fourier transform infrared spectroscopy (FTIR) was conducted using a FTIR spectrometer Shimadzu IRAffinity-1 at room temperature. Spectra were collected using KBr pellets in the spectral range 4000–500 cm^−1^, with the resolution of 4 cm^−1^.

### 2.3. In Vitro Antifungal Activity

*Candida albicans* ATCC 10231, *Candida albicans* SC5314 (ATCC MYA-2876) [22], *Candida parapsilosis* ATCC 22019, *Aspergillus fumigatus* ATCC 13073, *Trichophyton mentagrophytes* ATCC 9533 and *Microsporum gypseum* ATCC 24102 were used for evaluation of antifungal activity. *Candida* genus was grown in Sabouraud media (SAB; 4% glucose, 1% Bacto peptone, pH 5.6, 2 wt% agar to solidify) at 37 °C. The group of filamentous fungi was grown onto Potato dextrose agar plates (PDA) at 30 °C.

#### 2.3.1. Agar Diffusion Assay

Dried microspheres (0.25–1 mg) were placed on top of SAB plates inoculated with fungi and the inhibition zones were measured. For *C. albicans*, activity of microspheres was tested in the suspension as well, where 50 µL of suspension (5 mg/mL in PBS, pH 7.4) was added to SAB plates previously inoculated with fungal cells (10^5^ CFU/mL). Microspheres without polyenes and polyene stock solutions in DMSO (5 mg/mL) were used as controls.

#### 2.3.2. Broth Microdilution Assay

The minimum inhibitory concentration (MIC) of the tested compounds was tested by the standard broth microdilution assay, recommended by the European Committee on Antimicrobial Susceptibility Testing (EUCAST, Antifungal MIC method for yeasts, v 7.3.1) [23]. Briefly, fungal cells grown on YPD agar medium were diluted in liquid RPMI 1640 medium with 2% glucose to a concentration of 10^4^ cells/mL. The assay was performed in 96-well microtiter plates, employing serial twofold dilutions of the tested substances in a final volume of 200 µL. All compounds were tested in triplicate in a concentration range from 0.1–20 µg/mL. The MIC values were determined after 24 h or 48 h for dermatophytes of incubation at 37 °C without shaking by measuring the absorbance at 530 nm using a Tecan Infinite 200 Pro multiplate reader (Tecan Group Ltd., Männedorf, Switzerland). The negative (medium only) and positive (cells only) controls, incubated on the same plate, were used as references to determine the growth inhibition rate. Samples exhibiting growth inhibitory values of >90% were classified as active agents.

### 2.4. In Vivo Toxicity in Zebrafish

Experiments in zebrafish (*Danio rerio*) were carried out according to general rules of the OECD Guidelines for the Testing of Chemicals (OECD 2013). All experiments were performed in compliance with the European directive 2010/63/EU and ethical guidelines of the Guide for Care and Use of Laboratory Animals of the Institute of Molecular Genetics and Genetic Engineering, University of Belgrade. Embryos of wild type zebrafish were provided by Dr. Ana Cvejic (Wellcome Trust Sanger Institute, Cambridge, UK), raised to adult stage in a temperature- and light-controlled zebrafish facility at 28 °C, standard 14:10-h light-dark photoperiod and regularly fed with dry flake food twice a day and *Artemia nauplii* once daily. Zebrafish embryos produced by pair-wise mating were distributed into 24-well plates (10 embryos per well in 1 mL embryo water (0.2 g/L of Instant Ocean^®^ Salt in distilled water)) and raised at 28 °C. At 4–6 h post fertilization (hpf) embryos were treated with either PHA-microspheres or the microspheres with 20% (*w*/*w*) polyene content (0.2, 0.5, 1, 1.5 and 2 mg/mL) or free polyenes (1, 2, 5, 10, 20, 50, 100, 150 and 200 µg/mL). DMSO (0.25%) was used as negative control. Zebrafish embryos were treated by adding the obtained PHA/polyene microspheres in the form of suspension into the embryo water in which embryos were grown. Experiments were performed in triplicate using 30 embryos per treatment. Apical endpoints for toxicity evaluation (Supplementary File, Table S1) were recorded at 24, 48, 72, 96 and 120 hpf using an inverted microscope (CKX41, Olympus, Tokyo, Japan). Dead embryos were counted and discarded daily. At 120 hpf, embryos were inspected for heartbeat rate, anesthetized with 0.1 wt % tricaine solution, photographed and killed by freezing at −20 °C for ≥24 h.

### 2.5. Hepatotoxicity and Myelotoxicity

Transgenic *Tg*(*fabp10*:EGFP) zebrafish embryos with fluorescently labeled liver were exposed in the period from 72 to 120 hpf to different therapeutically relevant doses (½ × MIC-400 × MIC) of the tested samples. DMSO (0.125%, *v*/*v*) was used as a negative control. Hepatotoxicity was determined in comparison to the control group according to yolk consumption, liver color and liver area (LA) index. LA index was calculated as the ratio between liver area and embryonic lateral area [24,25], using ImageJ program.

Myelosupressive and inflammatory activity of the free polyenes and biopolymeric formulations was investigated using transgenic *Tg*(*mpx*:EGFP)i114 zebrafish embryos with fluorescently labeled neutrophils. Transgenic embryos were generated by natural spawning and reared in fish embryo water at 28 °C. At 26 hpf, embryos were exposed to different doses (½ × MIC to 400 × MIC) of the tested samples and inspected at 120 hpf stage under a fluorescence microscope (Olympus BX51) for the neutrophils presence and fluorescence intensity. Neutrophils occurrence was determined by measuring embryo’s fluorescence at excitation/emission wavelength of 488/509 nm using a Tecan Infinite 200 Pro multiplate reader and expressed in percentage of control (DMSO-treated group).

### 2.6. Zebrafish Infection Model of Disseminated Candidiasis

Wild-type zebrafish embryos and GFP expressing strain M137 of *C. albicans* [26] (provided by Prof Bernhard Hube, Department of Microbial Pathogenicity Mechanisms, Hans Knöll Institute, Jena, Germany) were used for infection experiments. To obtain log-phase cells for injection, single colonies of M137 strain from Yeast Extract–Peptone–Dextrose (YPD; 1% yeast extract, 2% peptone, 2% glucose and 2% agar to solidify) plates were inoculated in liquid YPD medium, grown overnight at 37 °C with shaking (180 rpm), sub-cultured 1:100 in the same medium and grown to the optical density OD_530_ of ~0.7–0.8. To prepare the final inoculum for microinjection, 2 mL of the cells were pelleted by centrifugation (1500× *g* for 10 min) (Centrifuge 5415D, Eppendorf, Hamburg, Germany), washed three times with sterile PBS and suspended in 5% polyvinylpyrrolidone (PVP) to achieve concentration of 2 × 10^7^ cells/mL. At 24 hpf, embryos were manually dechorionated and kept in embryo water at 28 °C. At 32–33 hpf, embryos were anesthetized with tricaine-methane sulfonate (200 µg/mL) and microinjected by a pneumatic picopump (PV820, World Precious Instruments, Sarasota, FL, USA) with ~5 nL of *C. albicans* M137 cells suspension through the otic vesicle into the hindbrain (i.e., 40–70 fungal cells per embryo). Infected embryos were kept at 28 °C and at 2 hpi treated with free polyenes (½ × MIC, 1 × MIC and 2 × MIC) and microspheres with 20% (*w*/*w*) polyene content (½ × MIC, 1 × MIC, 2 × MIC, 3 × MIC, 6 × MIC, 10 × MIC and 20 × MIC). Embryos injected with 5% PVP were used as the control group. Survival of infected fish was monitored at regular intervals up to 4 dpi under a stereomicroscope (SMZ143-N2GG, Motic, Vancouver, BC, Canada). Dead fish were removed daily. Experiments were repeated four times using 30 fish per treatment.

To determine fungal burden, individual fish alive at 2 and 4 dpi, were homogenized in Eppendorf tubes containing 200 µL 1×PBS supplemented with nalidixic acid (70 µg/mL) and ampicillin (100 µg/mL) using a pestle. Serial dilutions were made in 1×PBS with antibiotics and plated onto YPD medium. Plates were incubated at 37 °C for 3 days prior to counting. Filamentation and dissemination of *C. albicans* infection within embryos were examined at different time points (1–4 dpi) under a fluorescence microscope (Olympus BX51).

### 2.7. Statistical Analysis

Toxicological parameters (LC_50_—the dose killing 50% of the treated embryos, and EC_50_—the dose affecting 50% of the treated embryos) were determined by program ToxRatPro Version 2.10.05 (ToxRat Solution GmbH, Alsdorf, Germany) using probit analysis with linear maximum likelihood regression. Kaplan-Meier embryos survival curves were generated by software GraphPad Prism Version 6.01 (GraphPad, San Diego, CA, USA), statistically significant differences in embryos survival rates between the treated and untreated groups were determined using a Log rank (Mantel-Cox) test provided within software. Differences in heartbeat rate, LA index and the neutrophils occurrence between control and treated groups and between the groups treated with free polyenes and PHA/polyene formulations were determined using Student’s *t*-test using SPSS 20 (SPSS Inc., Chicago, IL, USA) software. Levels of statistical significance were denoted as: * *p* < 0.05, ** *p* < 0.01, *** *p* < 0.001.

## 3. Results

### 3.1. PHA/Polyene Microspheres Morphology and Characterization

**Figure 1:**
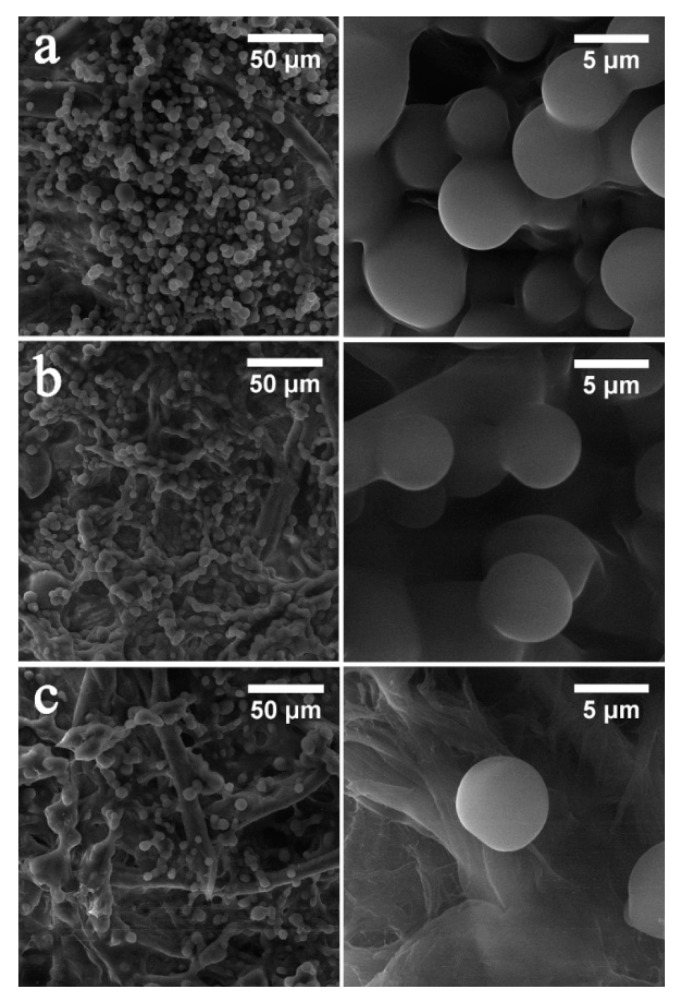
Morphology of pure biopolymer and polyene-containing biopolymer microspheres: (**a**) PHA; (**b**) PHA-20-Nys and (**c**) PHA-20-AmB at lower magnification (scale bar of 50 µM) and higher magnification (scale bar of 5 µM) are shown. Background structures are cellulose filaments of the paper on which samples were deposited.

Mcl-PHA/polyene microspheres were successfully produced by solvent evaporation technique from double emulsions to investigate their potential application as carriers for Nys and AmB. Uniform microspheres were obtained using both pure PHA and PHA/polyene mixture, and were denoted as PHA (pure biopolymer), PHA-10-Nys and PHA-20-Nys (loaded with 10 and 20%, *w*/*w* of Nys), and PHA-10-AmB and PHA-20-AmB (loaded with 10% and 20%, *w*/*w* of AmB) microspheres. SEM micrographs did not show obvious differences in size and morphology between PHA and PHA/polyene microspheres (Figure 1).

All samples appeared smooth and uniform, with diameter of microspheres between 7 and 8 µm indicating that the incorporation of polyenes into PHA did not affect microstructure of the microspheres (Figure 1). Incorporation of the polyenes was confirmed both visually and by FTIR analysis (Figure S1; [27,28,29,30]).

**Figure 2:**
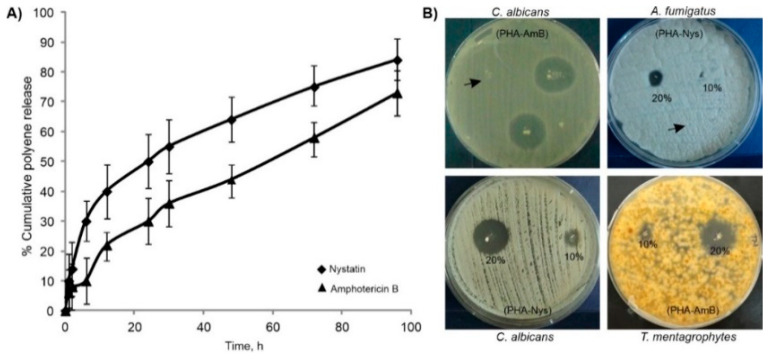
Cumulative release profiles of polyenes from PHA/polyene microspheres in phosphate buffer (**A**) and in vitro antifungal activity of dry PHA/polyene microspheres on agar plates (**B**). Representative plates showing inhibition zones of growth of the tested fungal pathogens around microspheres are shown. Arrow marks PHA microspheres without polyene.

Release of the polyenes from biopolymer-based microspheres with 20% polyene loading was investigated under dynamic conditions at 37 °C, using phosphate buffer as a release medium (Figure 2A). At the end of incubation period PHA-20-Nys and PHA-20-Amb samples released overall 81% and 70% of Nys and AmB, respectively. The microspheres with Nys released more the polyene, and at a higher rate during the early incubation period (time point 6 h). Both samples remained stable and preserved their structural integrity until the end of incubation period (data not shown).

### 3.2. In Vitro Evaluation of Antifungal Activity

To evaluate antifungal activity of the PHA/polyene formulations, agar diffusion assay was firstly used, and the diameter of growth inhibition zone was determined for each tested pathogen (Figure 2B, Table 1). *Candida* spp. were more susceptible compared to the filamentous fungi. The strongest activity was observed on *C. albicans* (8 and 20 mm for PHA-10-Nys and PHA-20-Nys, respectively; and 16 and 24 mm for 10% and 20% *w*/*w* PHA/AmB, respectively) and the weakest on *A. fumigatus* (0 and 8 mm for 10% and 20% PHA/Nys and 8 and 12 mm for PHA-10-AmB and PHA-20-AmB, respectively). For *C. albicans*, microsphere suspension showed a similar zone of inhibition (20 mm) to dried preparation and commercially available antifungal susceptibility disks (Figure S2) indicating that the microsphere suspension could also be used in infection studies. Additionally, the sensitivity of the fungal species to the free polyenes and biopolymer-based polyene formulations was investigated by determining the minimal inhibitory concentration (MIC) (Table 1). As dried PHA microspheres with 20% of polyenes were more active than those containing 10% of polyene, the MIC values were determined only for 20% PHA/polyene formulations. As expected, PHA-20-Nys and PHA-20-AmB showed less activity than pure drugs over 24-h treatment, which could be attributed to slower drug release over this period of time. Nevertheless, the results obtained in these assays show that antifungal activity was retained upon the drug loading into PHA microspheres and that the polyene-loaded microspheres were active against all the tested pathogens.

### 3.3. Loading into PHA-Microspheres Reduced Polyene-Associated Toxicity

In order to investigate whether PHA microspheres and PHA microspheres loaded with 20%, *w*/*w* of polyenes (PHA-20-AmB and PHA-20-Nys) are safe for human use and whether loading of these polyenes into PHA microspheres reduces adverse effects of the free drugs, we examined their toxicity in vivo using zebrafish (*Danio rerio*) embryos as a preclinical animal model system. Microspheres with 20% polyene loading were chosen as treatment based on the results of the in vitro antifungal experiments (stronger antifungal effect).

**Figure 3:**
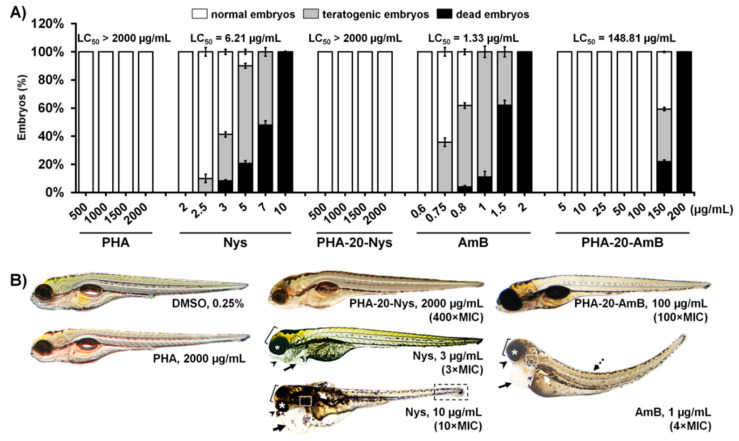
Polyene loading into PHA microspheres completely diminished inner organs toxicity caused by free polyene drugs. (**A**) Dose-dependent toxicity of the pure biopolymer (PHA), biopolymer-based polyene formulations (PHA-20-Nys and PHA-20-AmB) and free polyenes (Nys and AmB) in the zebrafish embryo model expressed as the LC_50_ value (the dose killing 50% of the treated embryos). (**B**) Morphology of the 120-hpf old zebrafish larvae following different treatments over a course of 5 days. Toxic side effects are indicated as: pericardial edema (black arrow), edema in the kidney region (white arrow), malformed head (bracket), jaw (arrowhead), eyes (asterisk), otoliths (white box), curved tail (dashed arrow) and necrotic tissue in the tail region (black box). At 10 µg/mL Nys (10 × MIC), only one out of 90 embryos survived by 120 hpf, and was seriously malformed and moribund.

Pure biopolymer microspheres were tested for potential systemic and inner organ toxicity, and after 5-days exposure no adverse effects have been observed up to concentration of 2000 µg/mL (Figure 3A). Further analyses revealed that neither PHA-20-Nys nor PHA-20-AmB have caused death, developmental malformations, cardiovascular dysfunctions (impaired heartbeat rate, heart morphology and body circulation) or signs of renal impairment in embryos exposed to the doses up to 2000 µg/mL and 100 µg/mL, respectively, indicating that the mcl-PHA/polyenes formulations are not toxic at 400-fold or 100-fold higher doses of the respective effective (MIC) doses on *C. albicans* (Figure 3 and Table 1). In contrast to this, the treatment with free polyenes (Nys and AmB) provoked inner organ toxicity and various developmental abnormalities in a dose-dependent manner followed by lethal outcomes. Zebrafish embryos have developed cardiac issues (pericardial edema and decreased heartbeat) when treated with 3 µg/mL of Nys (3 × MIC) and 0.8 µg/mL of AmB (3.2 × MIC), respectively (Figure 3B and Figure S3). The polyene-associated cardiotoxicity was particularly pronounced upon 1 µg/mL of AmB (4 × MIC) and was accompanied with serious skeletal malformations (microcephaly, malformed jaw and eyes, body deformation and tissue necrosis in the tail region) (Figure 3B). Apart from causing cardiovascular issues, AmB induced an appearance of edema in the kidney region of the fish exposed to the doses ≥1 µg/mL (≥4 × MIC), indicating its nephrotoxicity (Figure 3B). In addition, both drugs applied at the sub-therapeutic doses (<1 × MIC) reduced yolk absorption, indicating a possible hepatotoxicity of the applied treatments.

### 3.4. Hepatotoxicity Evaluation

To investigate the possible hepatotoxic effect of the free polyenes and biopolymeric formulations, transgenic *Tg*(*-2.8fabp10a*:EGFP) zebrafish embryos with fluorescently labeled liver were exposed to various doses of free polyenes and PHA-polyene formulations with 20% polyene content. During the period from 72 to 120 hpf (when liver is fully functional and metabolically active), changes in the liver area (LA) index, color and fluorescence representing the reliable hepatotoxicity endpoints were followed in real time [18,19].

**Figure 4:**
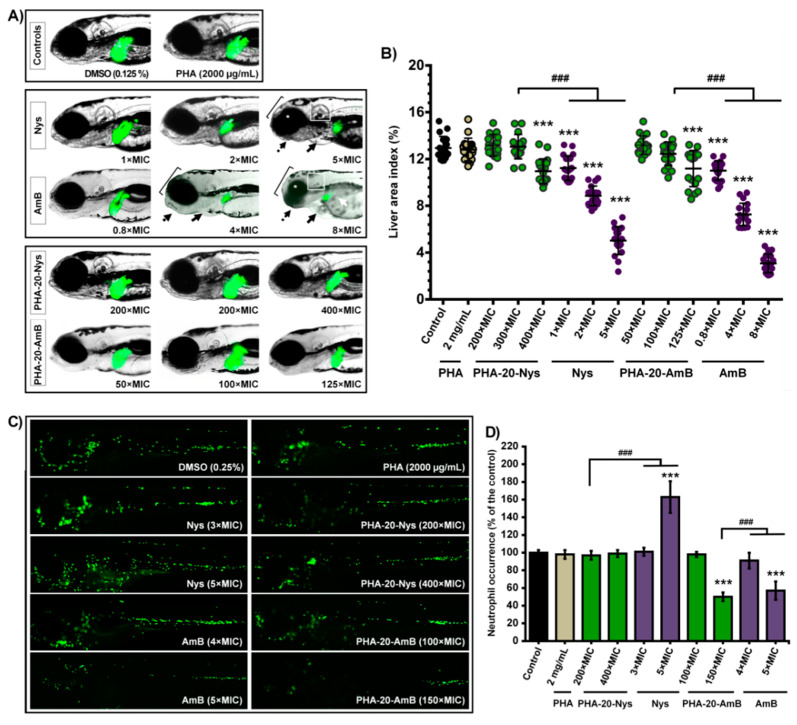
Dose-dependent hepatotoxicity (**A**,**B**) and myelotoxicity (**C**,**D**) of the pure biopolymer (PHA), free polyenes (Nys and AmB) and biopolymer-based polyene formulations (PHA-20-Nys and PHA-20-AmB). Changes in the liver size and fluorescence (**A**) and in the liver area index (**B**) as a function of the treatment type and concentration are shown. Hepatotoxicity endpoints (decreased liver area index, dark liver and reduced yolk consumption) have not been observed in the fish exposed to 2000 µg/mL of PHA, 1500 µg/mL of PHA-20-Nys (300 × MIC) and 100 µg/mL of PHA-20-AmB (100 × MIC). Conversely, free drugs Nys and AmB reduced the LA index at the therapeutic (1 × MIC, 1 µg/mL) and sub-therapeutic (0.2 µg/mL, 0.8 × MIC) doses (*p* < 0.001), respectively, as well as the yolk consumption at higher doses (white arrow). Additionally, free polyenes caused various skeletal deformities such as the malformation of head (bracket), jaw (dashed arrow), eyes (asterisk) and otoliths (boxed), and an appearance of pericardial edemas (arrow) at the higher therapeutic doses (≥4 × MIC for AmB and ≥5 × MIC for Nys). AmB was much more hepatotoxic, cardiotoxic and teratogenic then Nys. Distribution of the fluorescently labeled neutrophils upon different treatments (**C**) and the neutrophil occurrence in zebrafish embryos (**D**) are shown. Both polyenes were myelotoxic at the higher therapeutic doses, where AmB caused neutropenia (≥5 × MIC) and Nys inflammation (≥5 × MIC). On the other side, neither PHA nor polyene-loaded PHA microsphere caused the myelotoxic response. Statistically significant differences in the liver area index values between the control (DMSO) group and the treated groups (*** *p* < 0.001), as well as between the groups exposed to free polyenes and the respective polyene-loaded microspheres (### *p* < 0.001) are denoted.

At the stage of 120 hpf, we found that loading of both antifungal drugs into PHA microspheres drastically reduced liver toxicity when compared to the free drug treatments (Figure 4A, *p* < 0.001). In groups treated with free drugs, the LA index has already been reduced in some embryos at 0.2 µg/mL of AmB (0.8 × MIC) and 2 µg/mL of Nys (2 × MIC) when compared to the untreated group (Figure 4B) and gradually decreased in a dose-dependent manner (Figure S4). Cardiovascular issues (appearance of pericardial edema and heartbeat rate decrease) were also observed. In contrast to this, signs of hepatotoxicity have not been detected in any fish exposed to the doses up to 1500 µg/mL of PHA-20-Nys (300 × MIC) and 100 µg/mL of PHA-20-AmB (100 × MIC) (*p* > 0.1 for the LA index for both groups, Figure 4B). Moreover, half of the embryos treated with 125 µg/mL of PHA-20-AmB (125 × MIC) and 2000 µg/mL of PHA-20-Nys (400 × MIC) did not show any signs of hepatotoxicity, while the other half had to some degree lower LA index than the untreated fish (Figure 4B, *p* < 0.05).

### 3.5. Myelotoxicity Evaluation

Myelosupressive and inflammatory activity of the free polyenes and polymeric formulations was investigated using the transgenic *Tg*(*mpx*:EGFP)i114 zebrafish embryos with fluorescently labeled neutrophils. We found that neither PHA-20-Nys nor PHA-20-AmB caused neutropenia (neutrophil depletion) when applied at high doses of 2000 µg/mL (400 × MIC) and 100 µg/mL (100 × MIC), respectively (Figure 4C), indicating no immunotoxicity of the novel immobilized polyene formulations. Likewise, both Nys and AmB exhibited no toxicity towards neutrophils, but only when applied at much lower therapeutic doses than the drug-loaded microspheres (e.g., 3 × MIC). However, AmB µg/mL reduced neutrophil occurrence at 1 µg/mL (4 × MIC) by 43% compared to the control group (Figure 4D), while treatment with the doses of Nys ≥5 µg/mL (5 × MIC) increased neutrophils occurrence up to 173–273% and they were dispersed throughout the whole body of the treated fish, indicating the provoked tissues inflammation (Figure 4C,D).

### 3.6. Polyene-Loaded PHA Microspheres Are More Effective Than Free Polyenes against Disseminated C. albicans Infection In Vivo

**Figure 5:**
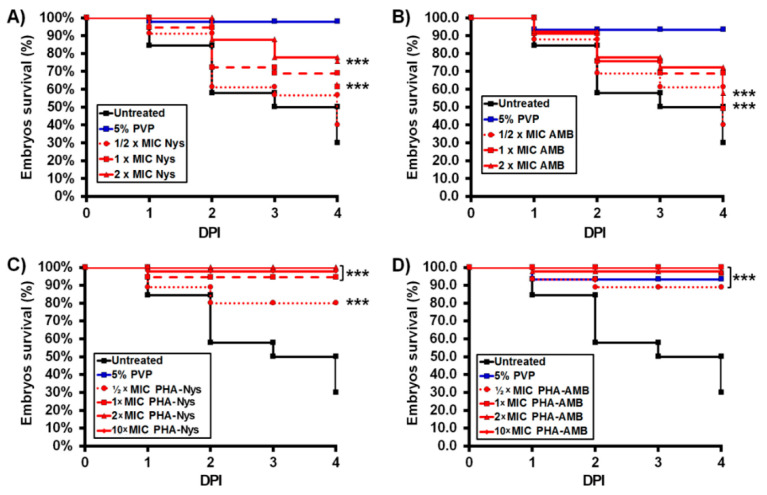
Treatment with PHA/polyene formulations rescued zebrafish embryos of the lethal *C. albicans* infection. Kaplan-Meier curves of the infected embryos survival upon the treatments with increasing doses of free polyenes (**A**,**B**) and the corresponding polyene-loaded microspheres (**C**,**D**) are shown. Embryos were monitored daily for the survival. Data are from four independent experiments using three replicate (*n* = 10 embryos/replicates) for each group. Statistically significant differences between the treated and the untreated groups were determined using a Log rank (Mantel-Cox) test (*** *p* < 0.001).

Antifungal efficacy of the novel PHA/polyene formulations was evaluated in the *C. albicans*-zebrafish embryo infection model [31,32]. Injection of 40–70 GFP-expressing cells of *C. albicans* into the hindbrain through the otic vesicle caused mortality of 70% of the untreated zebrafish embryos by 4 dpi (Figure 5A), with majority of embryo deaths occurring within the first 48 hpi. Embryos that survived by 3 dpi (10–20%) developed various deformities, such as scoliosis or pericardial edema and succumbed to the infection by 4 dpi (Figure S5).

Treatments with free polyenes (Figure 5A,B) and the corresponding polyene-loaded microspheres (Figure 5C,D) have significantly increased the survival of *C. albicans*-infected embryos when compared to the untreated group (*p* < 0.001). More importantly, the drug-loaded PHA microspheres displayed much higher antifungal efficacy than the free drugs. While only 76% and 58% of the infected embryos survived by 4 dpi upon the highest non-toxic concentration of Nys (2 × MIC, Figure 5A) and AmB (2 × MIC, Figure 5B), almost all infected fish (98–100%) were alive in the treatments with the same MIC doses of the corresponding PHA/polyene microspheres. Administration of the loaded microspheres even at the dose of 10 × MIC showed no adverse effect on *C. albicans*-infected embryos survival (97–100%) (Figure 5C,D).

**Figure 6:**
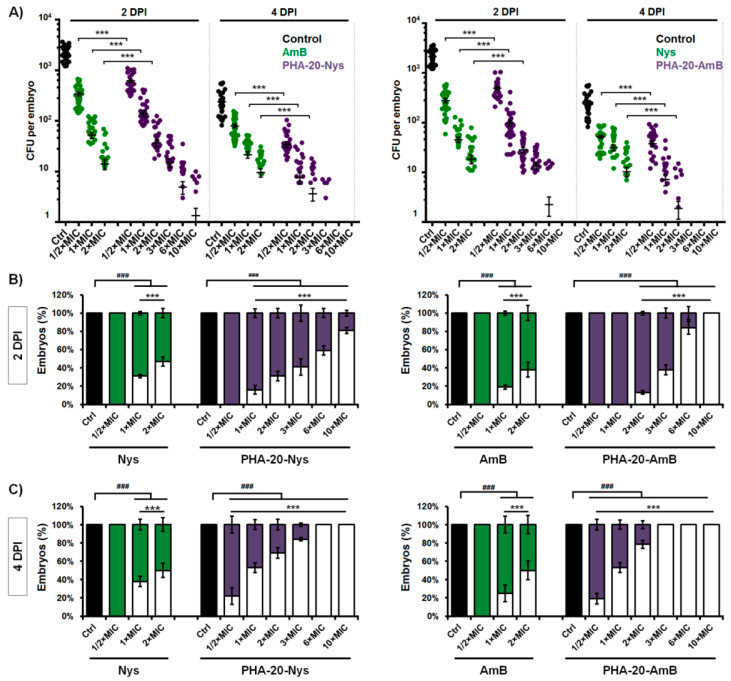
The polyene-loaded microspheres are more effective than the free polyenes in eradication of *C. albicans* infection. The dose- and time-dependent effect of the free polyenes and the polyene-loaded microspheres on the fungal burden (**A**) and the infection eradication (**B**,**C**) in the zebrafish infection model of disseminated candidiasis are shown. A) Fungal burden at 2 and 4 dpi is presented as *C. albicans* CFU per embryo, with each dot representing an individual fish (*n* = 32). The mean CFUs ± SD are shown. (**B**,**C**) Embryos with totally eliminated *Candida* (no CFU) are shown with empty bars, while those where the infection was detected are shown with solid bars. The presented data are a compilation of four independent experiments using eight embryos for each group (*n* = 32). Statistically significant differences between untreated and treated groups were determined using a Student’s *t*-test (### *p*< 0.001) and between free drug-treated and PHA/drug-treated groups using χ2 test (*** *p* < 0.001).

To investigate time- and dose-dependent effect of the applied treatments on fungal burden, the infected embryos were crashed on 2 and 4 dpi and plated on YPD media to count the remaining fungal cells (CFU). While *C. albicans* proliferated rapidly within the body of untreated embryos, reaching 1.4–3.7 × 10^3^ CFUs per embryo by 48 hpi, fungal burden in the embryos treated with the free drugs and the drug-loaded microspheres was significantly reduced in a dose-dependent manner (Figure 6A).

Free polyenes proved to be more efficient in eradication of *Candida* infection during first two days of the treatment (by 2 dpi) then the corresponding microspheres (*p* < 0.001). No fungal cells were detected (no CFU) in 47% and 37% of infected embryos upon the treatment with 2 × MIC of Nys and AmB, respectively, while only 31% and 13% of the fish receiving 2 × MIC dose of PHA-20-Nys or PHA-20-AmB had no infection (Figure 6A). In relation to the untreated group, the fungal burden in treated embryos that still harbored the infection at 2 dpi was reduced 2-3 log and stayed up to 6.9 × 10^1^ (2 × MIC Nys), 1.25 × 10^2^ (2 × MIC PHA-20-Nys), 7.9 × 10^1^ (2 × MIC AmB) and 8.2 × 10^1^ (2 × MIC PHA-20-AmB) (Figure 6A).

The percentage of embryos that were without *C. albicans* infection at 4 dpi was significantly higher in the groups exposed to the polyene-loaded microspheres then in the group exposed to pure polyenes (*p* < 0.001, for both groups). The treatments with 2 × MIC of AmB and Nys resulted only in a slight increase (~16%) in the number of infection-free embryos from 2 dpi to 4 dpi, with 47% and 53% of embryos harboring 7–40 CFU/fish and 9–31 CFU/fish at 4 dpi, respectively (Figure 6A,C). In contrast to this, the number of embryos with complete elimination of *Candida* infection was drastically increased from 2 dpi to 4 dpi upon the treatments with polyene-loaded microspheres-by 41% at 2 × MIC of PHA-20-Nys and by 56% at 2 × MIC of PHA-20-AmB) (*p* < 0.001), indicating a sustained drug release from the loaded microspheres. In these groups, only 22% and 31% of the fish remained infected harboring 6–18 CFU/fish and 2–15 CFU/fish, respectively (Figure 6A,C). However, the infection was completely eliminated by 4 dpi in all fish exposed to ≥3 × MIC of PHA-20-AmB and ≥6 × MIC of PHA-20-Nys (Figure 6C), indicating that a controlled release of polyenes could provide a complete eradication of *Candida* infection. It is important to note that treatments with high concentration of polyene/PHA microspheres (up to 20 × MIC, data not shown) did not induce any toxic effects and all infected larvae developed normally. In addition, the beneficial effect of the controlled drug release against fungal infection by 4 dpi was evident even at the sub-therapeutic doses of the polyene-loaded microspheres (½ × MIC), but not at ½ MIC of AmB or Nys.

### 3.7. Treatment with PHA-Polyene Microspheres Inhibits C. albicans Filamentation In Vivo

**Figure 7:**
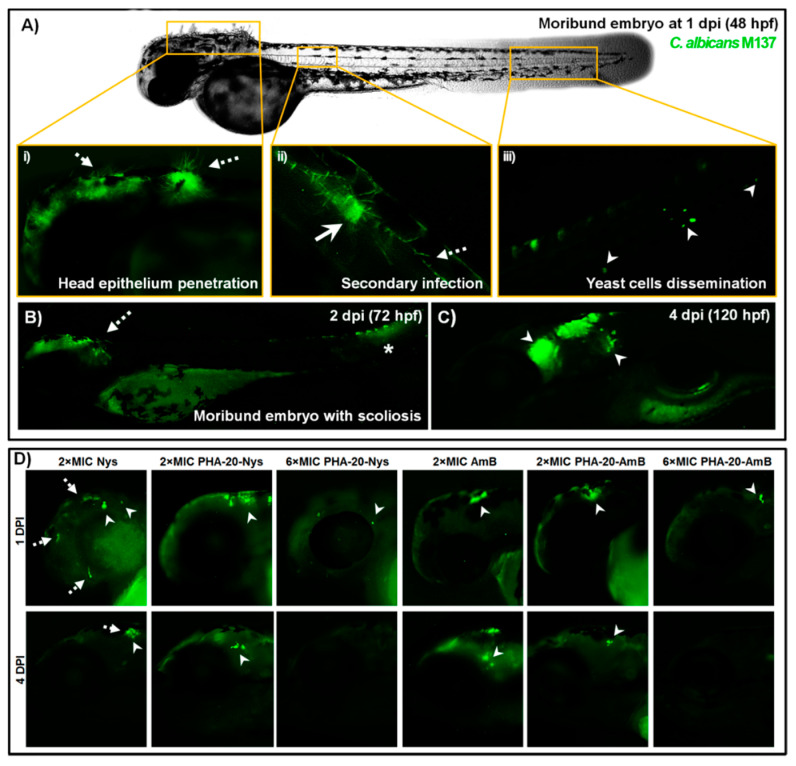
The treatments with the polyene-loaded microsphere led to the complete inhibition of *C. albicans* filamentation and prevented the infection dissemination. (**A**) The infection was developed in an untreated zebrafish embryo within 24 h following the injection of 40–70 of GFP-labeled cells of *C. albicans* (strain M137) into the hindbrain. During the first 24 h post infection (1 dpi), fungus filamented and penetrated the head epithelium (dashed arrow) (i), and disseminated into the caudal region forming the secondary foci of the infection (ii). The disseminated yeast-form cells (arrowhead) have also been detected at the end of the tail (iii). (**B**) Some of the untreated embryos that did not succumb the *Candida* infection by 1 dpi were malformed and moribund at 2 dpi (72 hpf), with the yeast-like structures present at the site of the body distortion (asterisk). Others that survived by 120 hpf had no visible malformations and the fungal infection mainly occurred within the head (arrowhead) (**C**). In contrast to this, fungal filamentation was successfully inhibited by 2 dpi upon the treatments with 2 × MIC doses of free polyenes, PHA-20-Nys and PHA-20-AmB, while *Candida* infection has not been detected at 4 dpi (120 hpf) in the fish treated with 6 × MIC dose of the polyene-loaded microspheres (**D**).

As filamentation represents one of the major virulence traits of *C. albicans* responsible for mucosal surfaces penetration and establishing localized and systemic infection, we analyzed the effect of applied treatments on fungal filamentation upon fluorescence microscopy at 24 h and 96 h post infection (treatment). The cells of *C. albicans* have filamented within the hindbrain of almost all untreated embryos a few hours after injection (Figure S5), and were disseminated throughout the body in both yeast and filamentous form, forming secondary foci of infection by 24 hpi (Figure 7A). Extensive hyphae were detected in all moribund embryos at 1 dpi, with numerous filaments penetrating head epithelium (Figure 7A(i)). Survival rate of the infected-untreated embryos was in high correlation with the level of fungus filamentation. In the portion of untreated embryos that did not succumb to *C. albicans* infection by 4 dpi, no filamentous structures were observed and yeast cells were mainly detected within the head (Figure 7C). In some cases, fungal mass occurred beneath protruded head epithelium (Figure S5), while in the embryos suffering from scoliosis a highly developed infection was detected at the site of body distortion (Figure 7B).

On the other side, the treatments with both polyene-loaded PHA microspheres and free polyenes efficiently inhibited *C. albicans* filamentation at each applied dose (Figure 7D). In contrast to vigorous yeast-to-hyphae transition in untreated embryos being realized a few hours post injection (Figure S5), neither filamentous structures nor germ tubes have been detected after 24-h treatments with 1 × MIC and 2 × MIC doses of free drugs and drug-loaded microspheres (Figure 7D). In PHA/polyene-treated embryos where the infection was still present at 4 dpi, fungus occurred only in a yeast-like morphology (Figure 7D) and its presence was additionally confirmed by the plating on YPD (Figure 6A). However, it is important to note that the fungal cells have not been detected in the treatments with ≥6 × MIC doses of polyene-loaded microspheres (Figure 7D), and these embryos developed without any visible side effect.

## 4. Discussion

Although Nys and AmB are regarded as generally safe for topical and oral application due to their poor absorption through skin, mucous membrane and gastrointestinal tract, systemic (parenteral) administration of both polyenes is largely limited because of their inner organs toxicity, particularly hepatotoxicity and nephrotoxicity [8,33]. Therefore, different formulations of polyenes (lipid-based preparations, liposomes, niosomes) were developed to modulate drug release, distribution and lower their toxicity and, subsequently, enable intravenous administration [34]. These drug delivery systems provide controlled release of active agent (in terms of desired location, release rate and duration) in order to achieve and maintain therapeutic concentration and avoid systemic toxicity [35]. Previous research has shown that scl-PHAs could be used as matrices for delivery of different bioactive agents (e.g., antibiotics [36,37], hormones [38] and anticancer drugs [39,40,41]). Immobilized or encapsulated systems in the form of micro-, nano-particles or films improved stability, release profiles and efficacy of active agents in biological systems. However, it was found that due to high crystallinity and hydrophobicity of the scl-PHA, surface pores were readily formed leading to fast drug release [12,13]. Bearing in mind low melting point and low crystallinity of the mcl-PHAs, more appropriate drug delivery matrices using this type of PHA could be obtained. In this study, we have successfully produced mcl-PHA-based polyene formulations in the form of microspheres. Microspheres have large surface area which makes them attractive for drug delivery applications. There was no obvious difference in size and surface morphology between pure biopolymer and biopolymer/polyene formulations (Figure 1), although it was expected that the incorporation of polyenes would affect the corresponding microsphere structure. We hypothesize that the addition of hydrophobic polyenes might have improved crystallization (packing) of the semi-crystalline mcl-PHA polymer chains and hence did not influence the diameter of polyene containing microspheres. Both formulations released polyenes in a sustained manner without burst release, Figure 2A. The higher total polyene release from samples with Nys (81 vs. 70% for PHA-20-Nys and PHA-20-AmB, respectively) could be explained by different drug distribution within the microsphere. Taking into account that this contribution to the cumulative release has happened early in the incubation, it could have originated from a portion of Nys molecules adsorbed onto microsphere surface or could be due to the structural differences between two polyenes. A system of conjugated double bonds in AmB might interact with PHA molecule more strongly than Nys and hence act as nucleating agent for biopolymer crystallization. Release of polyenes was confirmed in antifungal evaluation experiments. The obtained PHA/polyene microspheres exhibited excellent antifungal activity against the various fungal strains tested in this study (Figure 2B). These findings are in accordance with the results from our previous study where biopolymer/polyene formulations in the form of films were synthesized and evaluated [14].

In recent years, zebrafish has emerged as a highly predictive preclinical animal model for identification of pre-teratogenic and teratogenic drugs owing to their high molecular, genetic, physiological and immunological similarity to humans and good correlation in response to pharmaceuticals and bioactive compounds [42,43]. It is recommended by the FDA and the NIH and employed in the leading pharmaceutical companies for the drugs teratogenicity testing together with rodents, since rat and rabbit, as standard experimental animal models, failed to demonstrate birth defects of different drugs classes, such as thalidomide, warfarin, and coumarin [44,45], having significant consequences for a huge population of human newborns. In this study, we have comprehensively analyzed the toxicity profile of the novel polyenes/biopolymer formulations and the corresponding free drugs in order to determine the survival, developmental toxicity, cardiotoxicity, nephrotoxicity, hepatotoxicity and myelotoxicity of zebrafish embryos upon the applied treatments. While the free nystatin and amphotericin B were lethal in the zebrafish model in the dose-dependent manner (Figure 3A), which is in a line with previous studies on rodents [46], we found that polyenes incorporation into the biopolymer matrix has greatly reduced the toxic effects associated with both free drugs and remarkably improved the survival of treated fish.

Treatment with both PHA/polyene formulations did not cause death, malformation or inner organ toxicity even at exceptionally high doses (400 × MIC for PHA-20-Nys and 100 × MIC for PHA-20-AmB), while the treatment with the low doses of free polyenes (≥3 × MIC) caused severe developmental abnormalities and inner organ toxicity (Figure 3, Figure S4). Severity of the malformations observed in zebrafish embryos indicated that AmB is a more teratogenic drug than Nys. Besides being cardiotoxic, treatment with AmB also proved to be nephrotoxic (Figure 3B), which supports clinical findings where renal impairment represents a major clinical issue limiting parenteral and prolonged use of AmB [47]. Biopolymer/polyene formulations released antifungal agents at a slow rate and in a sustained manner, which prevented reaching toxic concentrations and subsequently adverse effects in zebrafish embryos.

Given that the liver injury is consistently reported as a serious issue associated with antifungal drugs [48,49], including parenteral application of Nys and AmB, we have investigated whether their loading into PHA microspheres could alleviate polyene-caused hepatotoxicity. Zebrafish model of liver toxicity is a proven accurate animal model for assessing drug-induced liver injury and has been previously shown superior to HepG2 assay for hepatotoxic drug identification [25,50]. We found that hepatotoxic effect has not been provoked even at high formulations doses (e.g., 300 × MIC for PHA-20-Nys and 100 × MIC for PHA-20-AmB), while pure dugs administered in soluble form (in DMSO) appeared to be hepatotoxic even at the sub-therapeutic dose (0.8 × MIC AmB) (Figure 4A,B). More importantly, both AmB and Nys exhibited primarily hepatotoxic effect over cardiotoxicity and developmental toxicity in the zebrafish model. These findings were in accordance with the results of the teratogenicity assay and correspond to the clinical implications in humans, and also can be explained by continual and slow release of the polyenes from PHA microspheres.

Innate immune system cells, particularly neutrophils, play a major role in the defense against *Candida* infections in both humans and zebrafish. As topical and oral drugs, neither Nys nor AmB are associated with systemic inflammation due to absence of absorption through skin and gastrointestinal tract. However, given that their intravenous application results in diverse toxicity reactions, the assessment of immunomodulatory effects of polyene formulations for potential parenteral administration is of a pivotal significance. We found that the treatments with neither PHA-20-Nys nor PHA-20-AmB have caused immunotoxicity in zebrafish embryos (Figure 4C,D). In contrast to this, the treatment with the free drugs has influenced the zebrafish immune system. Neutropenia was observed in embryos treated with 5 × MIC AmB (Figure 4D), while the treatment with Nys (≥5 × MIC) increased neutrophils occurrence (Figure 4C,D). Several studies have reported immunomodulatory effects of both AmB and Nys. Besides direct fungicidal activity, in vitro and in vivo studies have shown that AmB enhances phagocytic and antimicrobial activity of macrophages [51], which in turn inhibits or decreases chemotactic responsiveness, phagocytic activity and viability of human neutrophils [52]. Inflammation has previously been observed as an acute infusion-related adverse effect associated with the nystatin treatment, caused by the increased level of pro-inflammatory cytokines [53,54].

The model of disseminated candidiasis employed in this study has proven particularly useful to dissect host and pathogen disease determinants, enabling the tracking of yeast-to-hyphae transition, pathogen dissemination, early host immune response and the efficacy of applied anti-*Candida* agents [31,32,55,56]. As expected, the treatments with both free polyenes and the corresponding polyene-loaded microspheres have significantly increased the survival of *C. albicans*-infected embryos when compared to the untreated group (Figure 5A–D). The obtained results have shown rapid, but limited antifungal efficacy of the free polyenes, which can result in recurrent infection. On the other hand, drug-loaded PHA microspheres displayed much higher antifungal efficacy than the free drugs. The number of embryos with completely eliminated *Candida* infection from 2 dpi to 4 dpi was drastically increased in the treatments with polyene-loaded microspheres (by 41% at 2 × MIC of PHA-20-Nys and by 56% at 2 × MIC of PHA-20-AmB) when compared to the treatments with 2 × MIC free drugs (~16%, Figure 6C). Moreover, better therapeutic effect of the polyene-loaded PHA microspheres compared to the free polyenes on curing lethal *Candida* infection was evident even at the sub-therapeutic doses. The most important outcome of the therapy with polyenes-loaded microspheres is that life-threatening *C. albicans* infection was completely eradicated at the doses >3 × MIC of either PHA-20-Nys or PHA-20-AmB and the infected larvae were developed without toxic adverse effects. Even more, infected fish were normally developed at 10 × MIC of PHA/polyene formulations, which is attributed to the slow drug release. On the other side, treatment with the free drugs could not be applied at such high doses, since the infected larvae appeared to be more sensitive than uninfected larvae when exposed to ≥3 × MIC doses at 36 hpf onwards, developing teratogenic malformations by 2–4 days post treatment (Figure S6). This phenomenon could explain why survival rate of the infected embryos at 2 dpi upon the treatment with microspheres-containing polyenes was higher than upon the treatment with the same dose of the free polyenes, regardless of the fact that the free drugs were more efficient in *Candida* elimination.

Since yeast-to-hyphal switch is closely related to virulence in *C. albicans* using fluorescence microscopy, we have examined the effect of PHA-20-Nys treatment on fungal filamentation within the infected embryos. We found that sustained release of polyenes from PHA microspheres efficiently inhibited *C. albicans* filamentation at the therapeutic doses, while at the sub-therapeutic doses applied treatments modulated fungal cells germination/filamentation (Figure 7). Loading of polyenes into the PHA microspheres has drastically reduced toxicity, while improving therapeutic index (Ti) of both antifungal drugs. According to the LC_50_ values (Figure 3A and Table S1), PHA-20-AmB (LC_50_ = 135.63 µg/mL) and PHA-20-Nys microspheres (LC_50_ > 2000 µg/mL) showed 111-fold and 359-fold less toxicity in comparison to AmB (LC_50_ = 1.22 µg/mL) and Nys (LC_50_ = 5.57 µg/mL), respectively. Furthermore, the Ti of PHA-20-AmB and PHA-20-Nys formulations were 28- and 71-times higher than those of corresponding unbound drugs (Table S2). To date, the teratogenic effects on the fetus from the administration of polyenes and their formulations during pregnancy remain largely unknown, because they have not been explored in adequate and well-controlled studies in pregnant women [57,58]. The animal studies on rats, mice and rabbits failed to demonstrate a risk of Nys formulations-based therapy to fetus [45,58], and they were not even conducted for AmB and AmB-based formulations. Therefore, the toxicological data obtained in this study on the adverse effects associated with free polyenes therapy in the developing zebrafish embryo model warrant special attention due to possible detrimental outcome to fetal development. In summary, our results demonstrate that the loading of free polyenes into mcl-PHA microspheres presents an effective strategy to successfully combat lethal disseminated *Candida* infection and, at the same time, completely eliminate toxicity and teratogenicity associated with both drugs and allows their application even at exceptionally high doses. The obtained biopolymer-based polyene could be potentially used for parenteral administration. To the best of our knowledge, this is the first study demonstrating the efficacy of mcl-PHA/Nys or mcl-PHA/AmB antifungal therapy against disseminated candidiasis in vivo.

## Figures and Tables

**Table 1:** Antifungal activity of different PHA/polyene formulations against *C. albicans*, *C. parapsilosis*, *A. fumigatus*, *M. gypseum* and *T. mentagrophytes*, expressed as the diameter of inhibition zones (mm) and MIC values (µg/mL).

Sample	*C. albicans* ATCC 10231	*C. parapsilosis* ATCC 22019	*A. fumigatus* ATCC 13073	*M. gypseum* ATCC 24012	*T. mentagrophytes* ATCC 9533
Zones of inhibition (mm)					
PHA	0	0	0	0	0
PHA-10-Nys	8	6	0	8	6
PHA-20-Nys	20	16	8	14	14
PHA-10-AmB	20	14	8	8	10
PHA-20-AmB	24	20	12	15	18
MIC (µg/mL)					
PHA	>500	>500	>500	>500	>500
PHA-20-Nys	5	5	10	10	10
PHA-20-AmB	1	1	4	4	2
Nys	1	1	4	4	8
AmB	0.25	0.25	2	2	0.50

## Data Availability

The data presented in this study are available on request from the corresponding author.

## Supplementary Materials

The following supporting information can be downloaded at: https://www.mdpi.com/article/10.3390/pharmaceutics14040696/s1. Table S1: Lethal and teratogenic effects observed in zebrafish (*Danio rerio*) embryos at different hours post fertilization (hpf); Table S2: Therapeutic profiles of free polyenes and the corresponding loaded mcl-PHA microspheres; Figure S1: FTIR spectra of the PHA, free polyenes and PHA-10-Nys (a) and PHA-10-AmB (b). Figure S2: Comparison of in vitro antifungal activity of three different formulations of AmB against *C. albicans*: (a) dried microspheres preparation containing 20% *w*/*w* AmB (250 µg (50 µg of AmB)); (b) microspheres suspension 25 mg/mL (250 µg microspheres (50 µg of AmB)) and (c) antifungal susceptibility disc (100 µg of AmB); Figure S3: Heart beating rate in the 120-hpf old zebrafish larvae exposed to different doses of the mcl-PHA microspheres, free drugs AmB and Nys, and PHA microspheres containing 20% polyenes in a period from 6 to 120 hpf; Figure S4: Dose-dependent hepatotoxicity of free drugs Nys and AmB addressed in the transgenic *Tg*(*fabp10*:EGFP) zebrafish larvae with fluorescently labeled liver. The embryos have been exposed to drugs in the period from 72 hpf to 120 hpf. Upon treatment with 8 × MIC and 12 × MIC of AMB, embryos exhibited delay in growth with large pericardial edema, completely non-resorbed necrotic (dark) yolk and weak or no liver fluorescence; Figure S5: Dissemination of *C. albicans* infection throughout the head a few hours after injection of yeast cells into the hindbrain. The yeast-form cells (arrowhead) filamented inside the hindbrain, formed hyphae (arrow) disseminating throughout head, some of which penetrated out the head epithelium (dashed arrow) by 12-16 hpi; Figure S6: Fungal infection makes the infected embryos more sensitive to polyene treatment. At 2 dpi, the embryos infected with *C. albicans* were more sensitive than control (5% PVP-injected) to 3 × MIC of AmB, failed to completely eliminate fungal infection (arrow) and developed teratogenic malformations—malformed jaw (arrowhead), scoliosis (dashed arrow) and lack of swim bladder (boxed). In contrast to this, *C. albicans*-infected embryos treated with 3 × MIC PHA-20-AmB was normally developed and fungal infection was completely eradicated.
