# Supplementary material for: Polyenes in Medium Chain Length Polyhydroxyalkanoate (mcl-PHA) Biopolymer Microspheres with Reduced Toxicity and Improved Therapeutic Effect against Candida Infection in Zebrafish Model
Source: Pharmaceutics. 2022 Mar 24;14(4):696. doi: 10.3390/pharmaceutics14040696 (PMC9028145; doi:10.3390/pharmaceutics14040696)
Supplement: Supplementary file 1 [file pharmaceutics-14-00696-s001.zip › pharmaceutics-1629535-supplementary.pdf]

pharmaceutics

Supplementary Materials: Polyenes in Medium Chain Length Polyhydroxyalkanoate (mcl-PHA) Biopolymer Microspheres with Reduced Toxicity and Improved Therapeutic Effect Against *Candida* Infection in Zebrafish Model

Aleksandar Pavic, Zoran Stojanovic, Marina Pekmezovic, Đorđe Veljović, Kevin O'Connor, Ivana Malagurski and Jasmina Nikodinovic-Runic

Table S1. Lethal and teratogenic effects observed in zebrafish (*Danio rerio*) embryos at different hours post fertilization (hpf).

Category	Developmental endpoints	Exposure time (hpf)				
		24	48	72	96	120
**Lethal effect**	Coagulated eggs [a]	●	●	●	●	●
	Lack of the heart beating	●	●	●	●	●
**Teratogenic effect**	Malformation of head	●	●	●	●	●
	Malformation of eyes [b]	●	●	●	●	●
	Malformation of sacculi/otoliths [c]	●	●	●	●	●
	Malformation of chorda	●	●	●	●	●
	Malformation of tail [d]	●	●	●	●	●
	Scoliosis	●	●	●	●	●
	Yolk edema	●	●	●	●	●
	Yolk deformation	●	●	●	●	●
	Growth retardation [e]		●	●	●	●
	Hatching			●	●	●
**Hepatotoxicity**	Yolk absorption			●	●	●
	Liver darkening			●	●	●
**Cardiotoxicity**	Pericardial edema		●	●	●	●
	Heart morphology			●	●	●
	Heart beating rate (beat/min)				●	●

a No clear organs structure is recognized; b Malformation of eyes was recorded for the retardation in eye development and abnormality in shape and size; c Presence of none, one or more than two otoliths per sacculus, as well as reduction and enlargement of otoliths and/or sacculi (otic vesicles); d Tail malformation was recorded when the tail was bent, twisted or shorter than to control embryos as assessed by optical comparation; e Growth retardation was recorded by optical comparison with the control embryos in a body length (after hatching, at and onwards 72 hpf) using an inverted microscope (CKX41; Olympus, Tokyo, Japan).

Table S2. Therapeutic profiles of free polyenes and the corresponding loaded mcl-PHA microspheres.

Sample	MIC	LC_{50}[a]	EC_{50}[b]	T index [c]	Ti [d]
		(µg/mL)		(LC_{50}/EC_{50})	(EC_{50}/MIC)
**Nys**	1.0	6.2	3.4	1.84	3.4
**PHA-20-Nys**	5.0	>2000	>2000	1.00	>400
**AmB**	0.25	1.3	0.8	1.71	3.1
**PHA-20-AmB**	1.0	148	135	1.10	135

a LC50 – the dose killing 50% of the treated embryos; b EC50 – the dose affecting 50 % of the treated embryos; c T index – toxicity index; d Ti – therapeutic index. The toxicological parameters (LC_{50}, the dose killing 50% of the treated embryos, and EC_{50}, the dose affecting 50 % of the treated embryos, values) were determined by the program ToxRatPro (ToxRat, Software for the Statistical Analysis of Biotests, ToxRat Solution GmbH, Alsdorf, Germany, Version 2.10.05) using probit analysis with linear maximum likelihood regression.

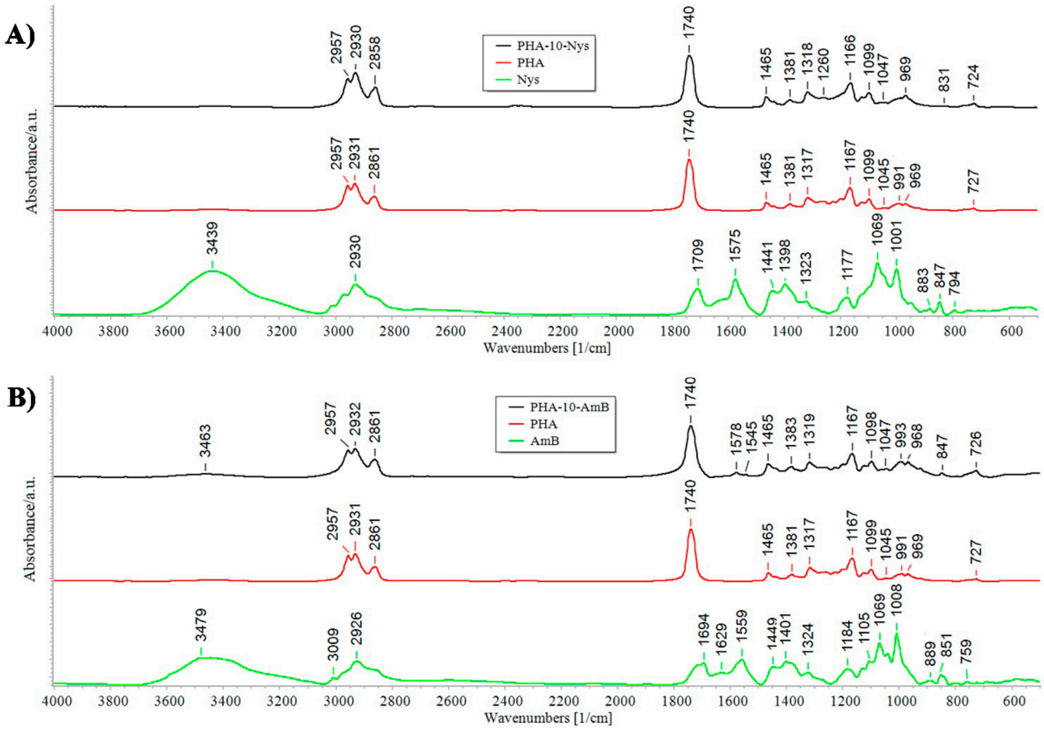

Figure S1. FTIR spectra of the PHA, free polyenes and PHA-10-Nys (**A**) and PHA-10-AmB (**B**).

FTIR spectra of the PHA, free polyenes and PHA/polyene formulations are presented in Fig. S1. FTIR spectrum of the PHA microspheres presented bands characteristic for PHA biopolymers: ester carbonyl group (C=O) stretching at 1740 cm^{-1}, CH_3, CH_2 and CH bending in the 1500-1300 cm^{-1}, C-O-C stretching in the 1300-1000 cm^{-1}, C-C backbone stretching in the 1000-800 cm^{-1} range and asymmetric $–CH_3$ and symmetric $–CH_2$- vibrations between 2800-3000 cm^{-1} (Fig. S1) [27]. Free polyenes exhibited characteristic broad band at 3439 (overlapping of N-H and O-H stretching vibrations), 2930 ($-CH_2$- stretching vibrations), 1709 (C=O stretching vibrations) and 1575 cm^{-1} (overlapping N-H bending and C=O stretching) for Nys [28] and at 3479 (N-H and O-H stretching), 2926 (CH_2, CH_3 asymmetric and symmetric stretching vibrations, CH stretching), 1694 (asymmetric stretching $–COO^-$), 1559 ($-NH_3^+$ symmetric bending coupled with $–COO^-$ asymmetric stretching), 1449 (CH_2, CH_3 asymmetric bending), 1401 (symmetric stretching vibrations $–COO^-$), 1184 (C-O-C asymmetric stretching vibrations), 1069 cm^{-1} (C-O asymmetric stretching) and at 889 cm^{-1} (-CH, $-CH_3$ bending) for AmB [29]. Following polyene incorporation into the biopolymer, FTIR spectra of the PHA/polyene microspheres showed predominantly bands characteristic for the PHA indicating efficient incorporation of hydrophobic polyenes into the polyester matrix [30]. Only the spectrum of formulations with AmB, exhibited AmB characteristic peaks at 3463 and 1578 cm^{-1}, indicating presence of the polyene and potential molecular interactions, especially via OH and N-H groups.

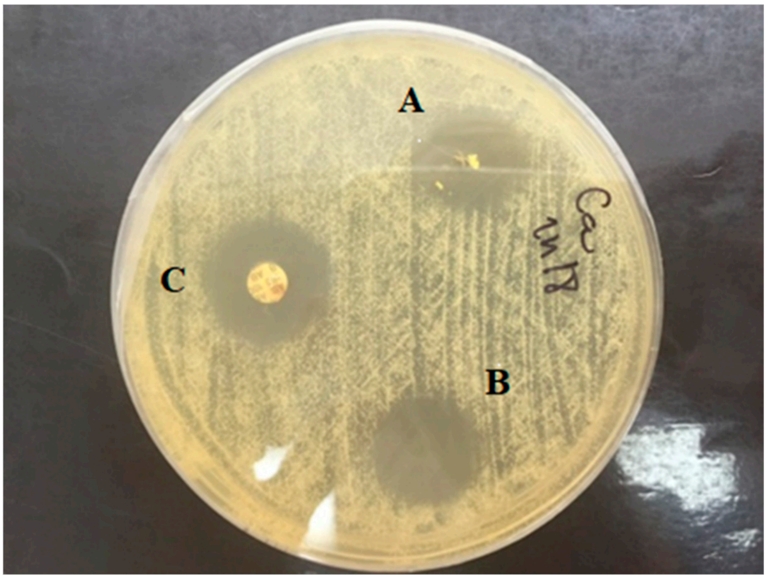

Figure S2. Comparison of *in vitro* antifungal activity of three different formulations of AmB against *C. albicans*: (**A**) dried micelle preparation containing 20% w/w AmB (250 μg of micelles /50 μg of AmB); (**B**) micelle suspension 25 mg/mL (250 μg micelles /50 μg of AmB)) and (**C**) antifungal susceptibility disc (100 μg of AmB).

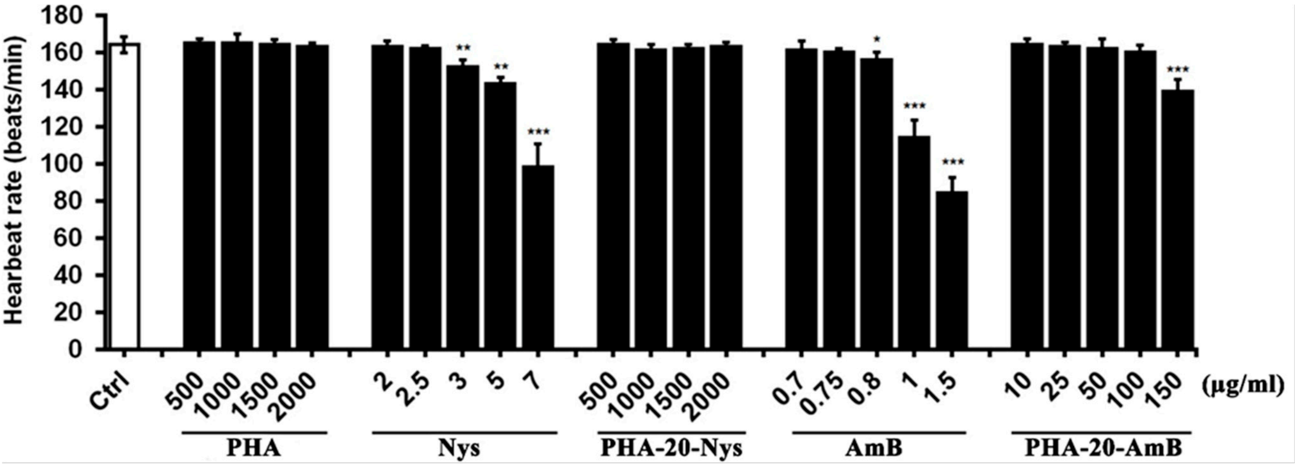

Figure S3. Heart beating rate in the 120-hpf old zebrafish larvae exposed to different doses of the mcl-PHA micelles, free drugs AmB and Nys, and PHA micelles containing 20% polyenes in a period from 6 to 120 hpf. * $p < 0.1$; ** $p < 0.01$; *** $p < 0.001$.

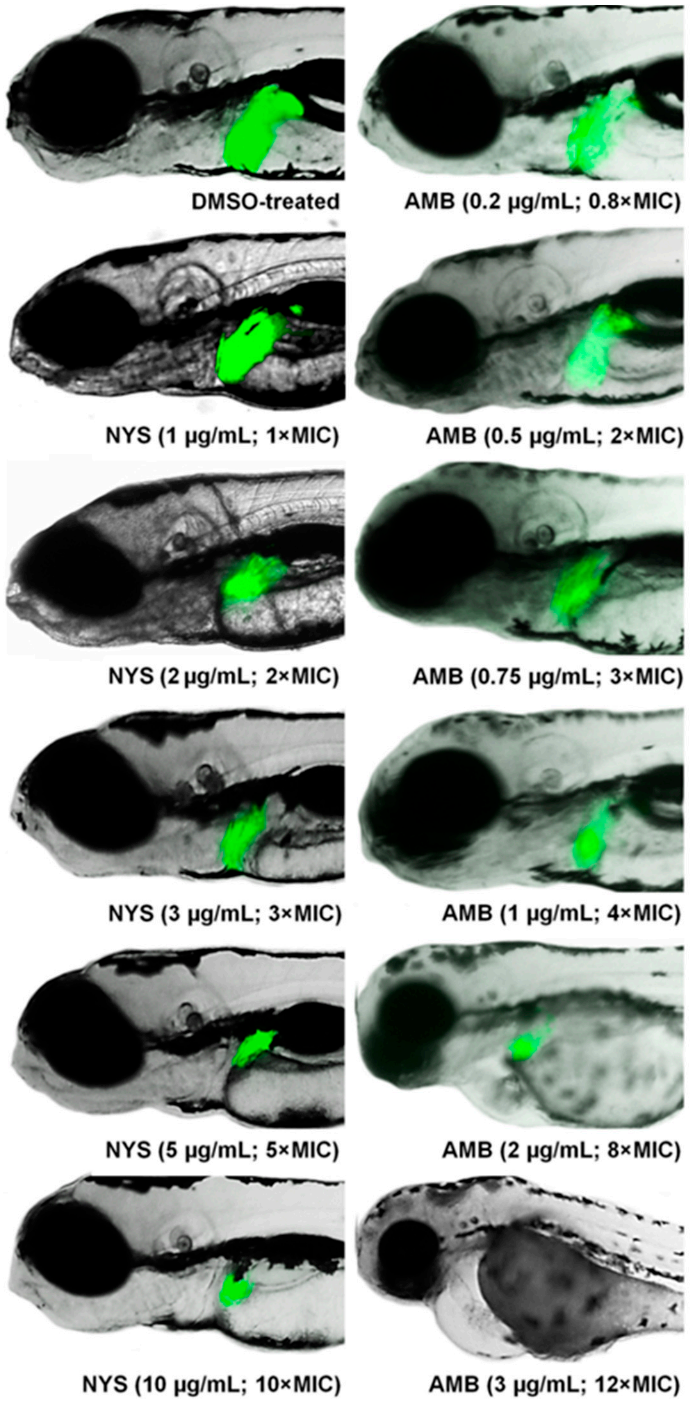

Figure S4. Dose-dependent hepatotoxicity of free drugs Nys and AmB addressed in the transgenic *Tg*(*fabp10*:EGFP) zebrafish larvae with fluorescently labeled liver. The embryos have been exposed to drugs in the period from 72 hpf to 120 hpf. The applied polyenes caused the liver size decrease in a dose-dependent manner. Upon the treatments with 8×MIC and 12×MIC of AMB, embryos exhibited delay in growth with large pericardial edema, completely non-resorbed necrotic (dark) yolk and weak or no liver fluorescence.

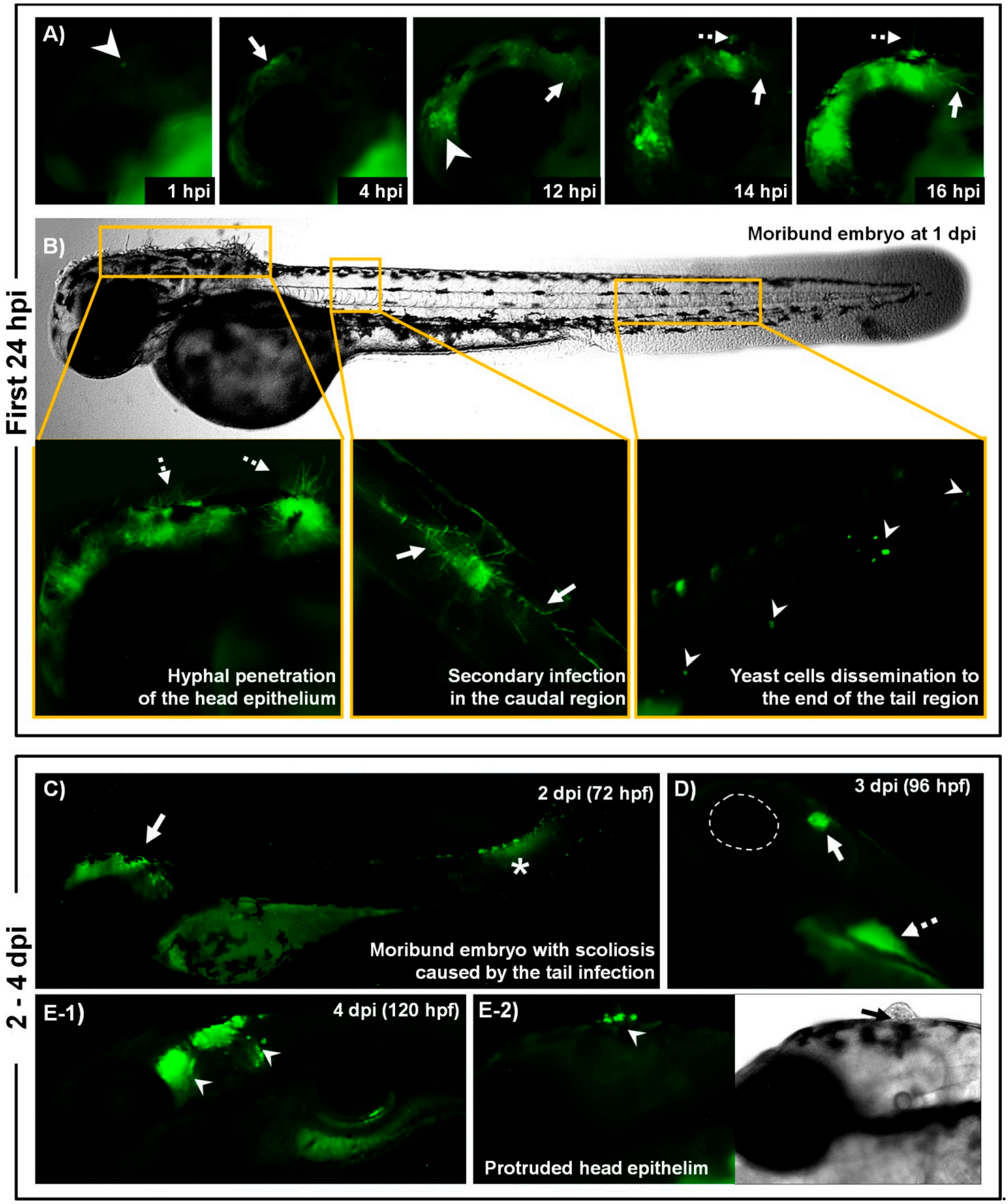

Figure S5. Dissemination of *C. albicans* infection in alive infected fish at different developmental stages without treatment. (**A**) A few hours after microinjection, the yeast-form cells (arrowhead) formed hyphae (arrow) inside the hindbrain and disseminated throughout the head, some of which penetrated out the head epithelium (dashed arrow) by 12-16 hpi, leading to the lethal outcome by 24-48 hpf. In some infected embryos, disseminated fungus formed secondary foci of infection (**B**) being visible as filaments in the caudal region and the yeast cells at the end of the tail. While some infected fish were malformed and moribund by 72 hpf (**C**), having the fungal infection within the brain (arrow) and at the site of the body distortion (asterisk), other that survived by 96 hpf (**D**) and 120 hpf (**E**) had no visible malformations, and the fungal infection (yeast cells) mainly occurred within intestine (dashed arrow) (**D**) and the head (arrow) (**E1**). In some fish, the fungus protruded the head epithelium without penetrating it (**E2**).

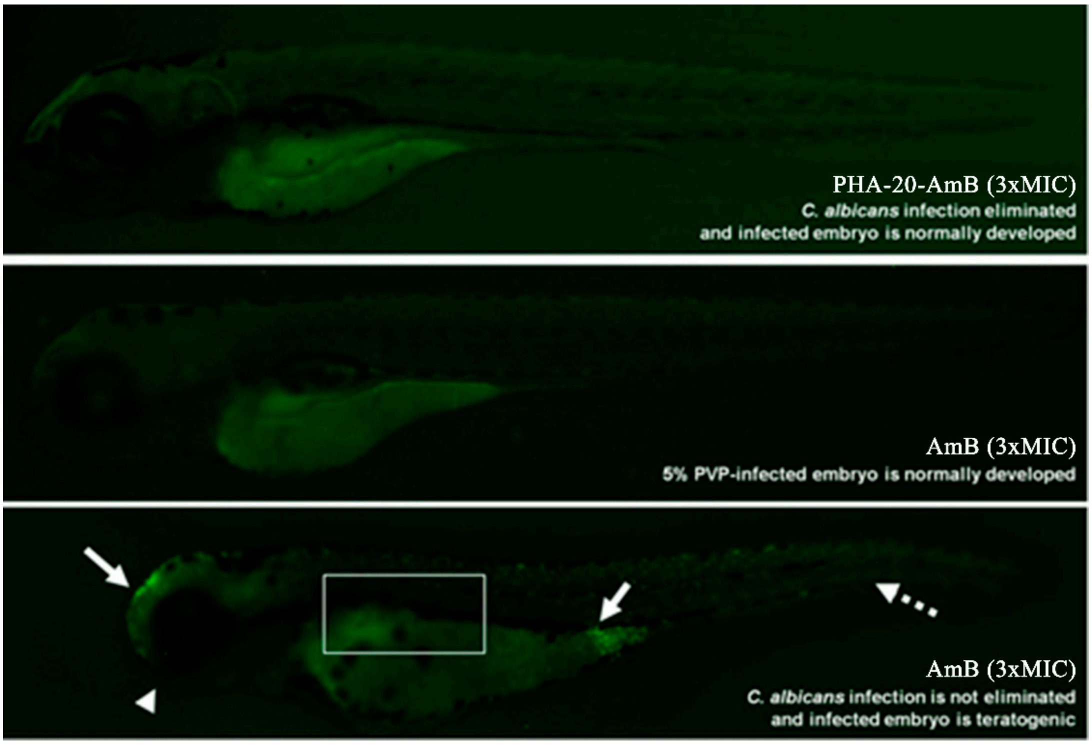

Figure S6. Fungal infection makes the infected embryos more sensitive to polyene treatment. At 2 dpi, the embryos infected with *C. albicans* were more sensitive than control (5% PVP-injected) to 3×MIC of AmB, failed to completely eliminate fungal infection (arrow) and developed teratogenic malformations – malformed jaw (arrowhead), scoliosis (dashed arrow) and lack of swim bladder (boxed). In contrast to this, *C. albicans*-infected embryos treated with 3×MIC PHA-20-AmB was normally developed and fungal infection was completely eradicated.

References:

27. Larrañaga, A.; Fernández, J.; Vega A.; Etxeberria, A.; Ronchel, C.; Adrio, J.L.; Sarasua, J.R. Crystallization and its Effect on the Mechanical Properties of a Medium Chain Length Polyhydroxyalkanoate. *J Mech Behav Biomed Mater* **2014**, 39, 87-94, doi.org/10.1016/j.jmbbm.2014.07.020
28. Mohammadi, G.; Namadi, E.; Mikaeili, A.; MOhamadi, P.; Adibkia, K. Preparation and Physicochemical Characterization of the Nystatin-loaded Eudragit RS100/PLGA Nanoparticles and Evaluation of their Anti-fungal Properties against *Candida albicans*. *J Drug Deliv Sci Technol* **2017**, 38, 90-96, doi.org/10.1016/j.jddst.2017.02.004
29. Gagos, M.; Arczewska, M. Spectroscopic Studies of Molecular Organization of Antibiotic Amphotericin B in Monolayers and Dipalmitoylphosphatidylcholine Lipid Multibilayers. *Biochim Biophys Acta* **2010**, 1798, 2124-2130, doi.org/10.1016/j.bbamem.2010.07.037
30. Salevic, A.; Stojanovic, D.; Levic, S.; Pantic, M.; Djordjevic, V.; Pesic, R.; Bugarski, B.; Pavlovic, V.; Uskokovic, P.; Nedovic, V. The Structuring of Sage (*Salvia officinalis* L.) Extract-Incorporating Edible Zein-Based Materials with Antioxidant and Antibacterial Functionality by Solvent Casting versus Electrospinning. *Foods* **2022,** 11, 390. doi.org/10.3390/foods11030390
